# Recurrent Bilateral Basilar Joint Subluxation in a Teenage Boy

**DOI:** 10.1016/j.jhsg.2021.07.001

**Published:** 2021-08-19

**Authors:** Thomas G. Knoedler, Kevin M. Condit, Stefan V. Zachary

**Affiliations:** ∗ Department of Orthopedics, University of Wisconsin Health, Madison, WI

**Keywords:** Dislocation, Stability, Subluxation, Thumb, Tightrope

## Abstract

Carpometacarpal (CMC) joint instability may be caused by either joint trauma or systemic ligamentous laxity in a setting of connective tissue disorders. Bilateral CMC joint dislocation is extremely rare and has only been described in 2 cases, both resulting from high-energy mechanisms in adults. Here, we present a case of recurrent, bilateral CMC joint subluxation and dislocation resulting from low-energy mechanisms in a pediatric patient with no diagnosable connective tissue disorder. Over a course of 4 years, the patient underwent 10 procedures, including bilateral closed reduction and immobilization, bilateral closed reduction and percutaneous pinning, bilateral tightrope placement, and eventual bilateral tightrope revision with anterior oblique ligament reconstruction. To date, the optimal treatment options for bilateral, low-energy CMC dislocations have not been well described, and these depend on the time from injury to closed reduction as well as postreduction joint stability. Tightrope placement and ligamentous reconstruction may be required in a setting of long-term instability.

Thumb opposition is paramount to hand function, and the first carpometacarpal (CMC) joint is vital for this process. Instability of the CMC may occur secondary to either systemic ligamentous laxity, such as in Marfan syndrome or Ehlers-Danlos syndrome, or joint trauma.

To our knowledge, there are only 2 reported cases of bilateral thumb CMC dislocation in the English-language medical literature. However, both were acute injuries occurring in adults with high-energy mechanisms: a motor vehicle collision while gripping the steering wheel and a motorcycle collision while gripping the handlebars.^1^^,^^2^ Here, the authors presented a rare case of recurrent episodes of subluxation and dislocation of bilateral CMC joints via low-energy mechanisms in a skeletally immature individual with no diagnosable connective tissue disorder.

## Case Report

**Figure 1:**
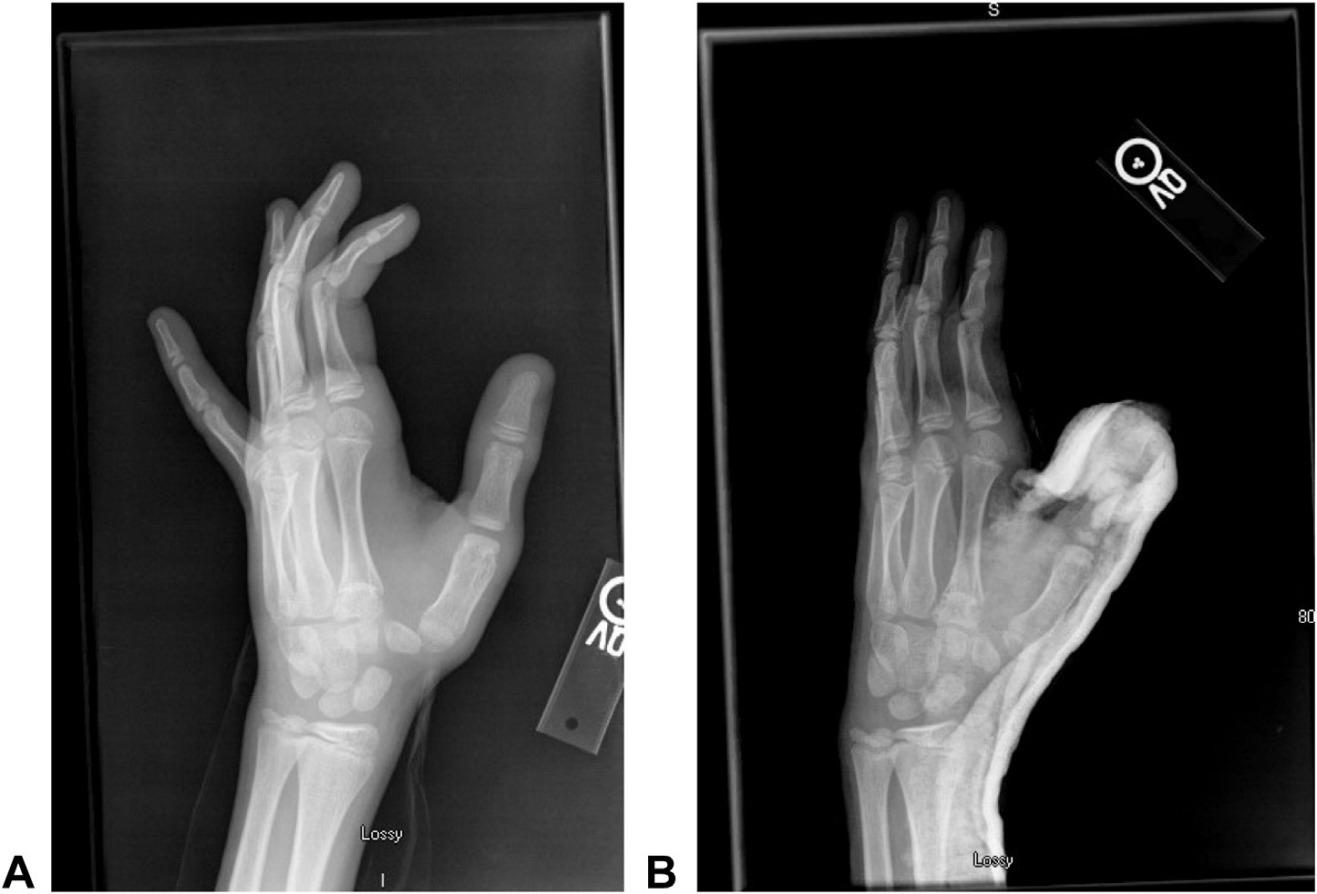
March 2011: Closed reduction of left CMC. The images demonstrate the **A** prereduction and **B** postreduction alignments of the CMC with a postreduction spica cast in place.

The patient was a 14-year-old boy who, prior to presentation at a hand clinic, had dislocated each of his CMC joints twice via low-energy mechanisms and had been treated with closed reduction and orthosis fabrication, followed by casting, at an outside institution. The representative radiographs of 1 closed reduction are shown in Figure 1. Following each episode of casting, he had an interval period of maintained reduction, followed by another dislocation.

**Figure 2:**
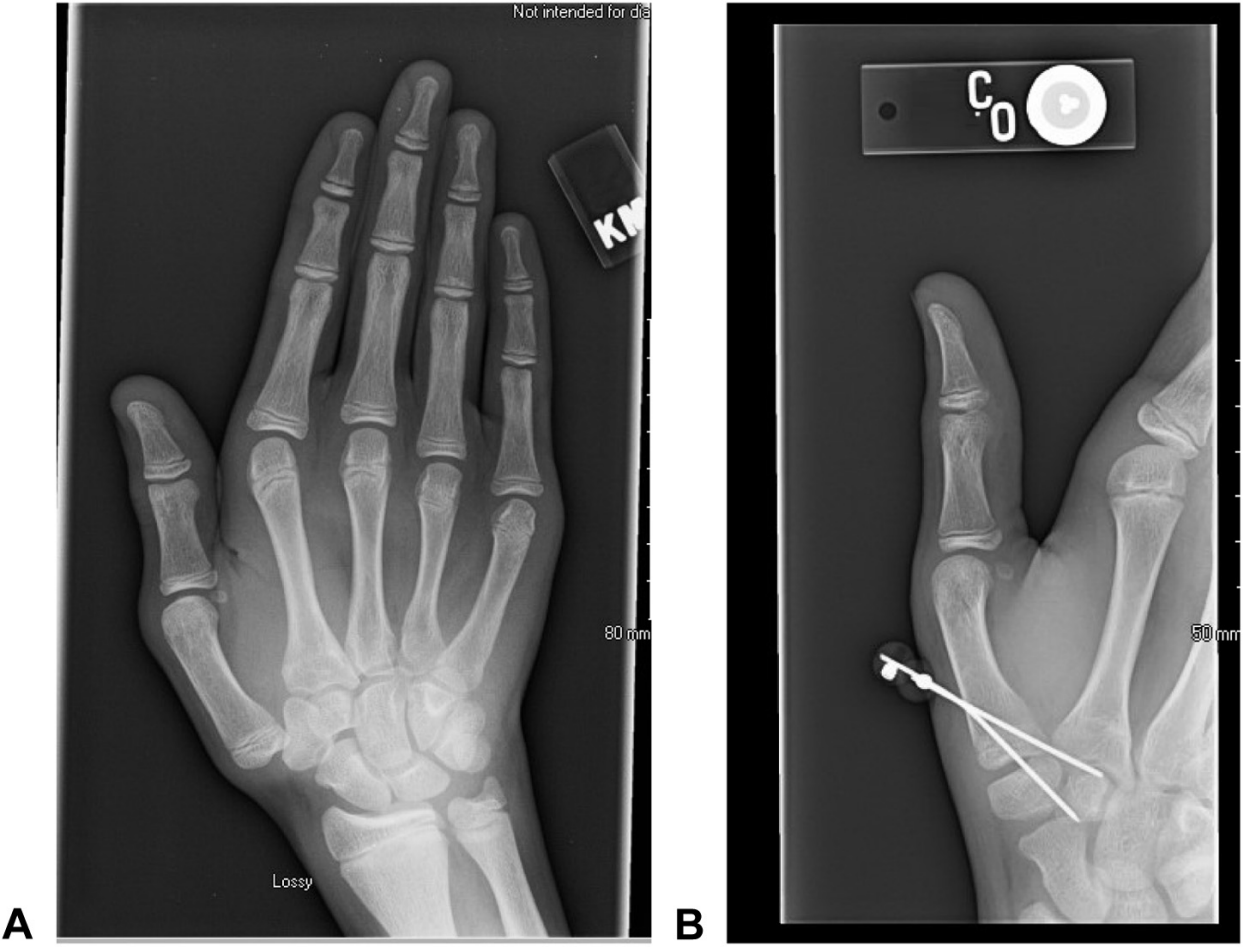
October 2013: Right CMC CRPP. The images represent the **A** preoperative and **B** postoperative alignments following the right CMC’s CRPP.

Following his third overall CMC dislocation (second right-sided dislocation), he was referred to our institution, and he presented to the hand clinic in October 2013. Because of the repetitive nature of his injury, along with prior literature showing good outcomes of less invasive interventions in the pediatric population, the decision to perform closed reduction and percutaneous pinning (CRPP) of the right thumb (Fig. 2) rather than to proceed with a more invasive procedure, such as ligament reconstruction (LR), was made. He tolerated the procedure well, and at the 1-month postoperative follow-up, his pins were removed.

**Figure 3:**
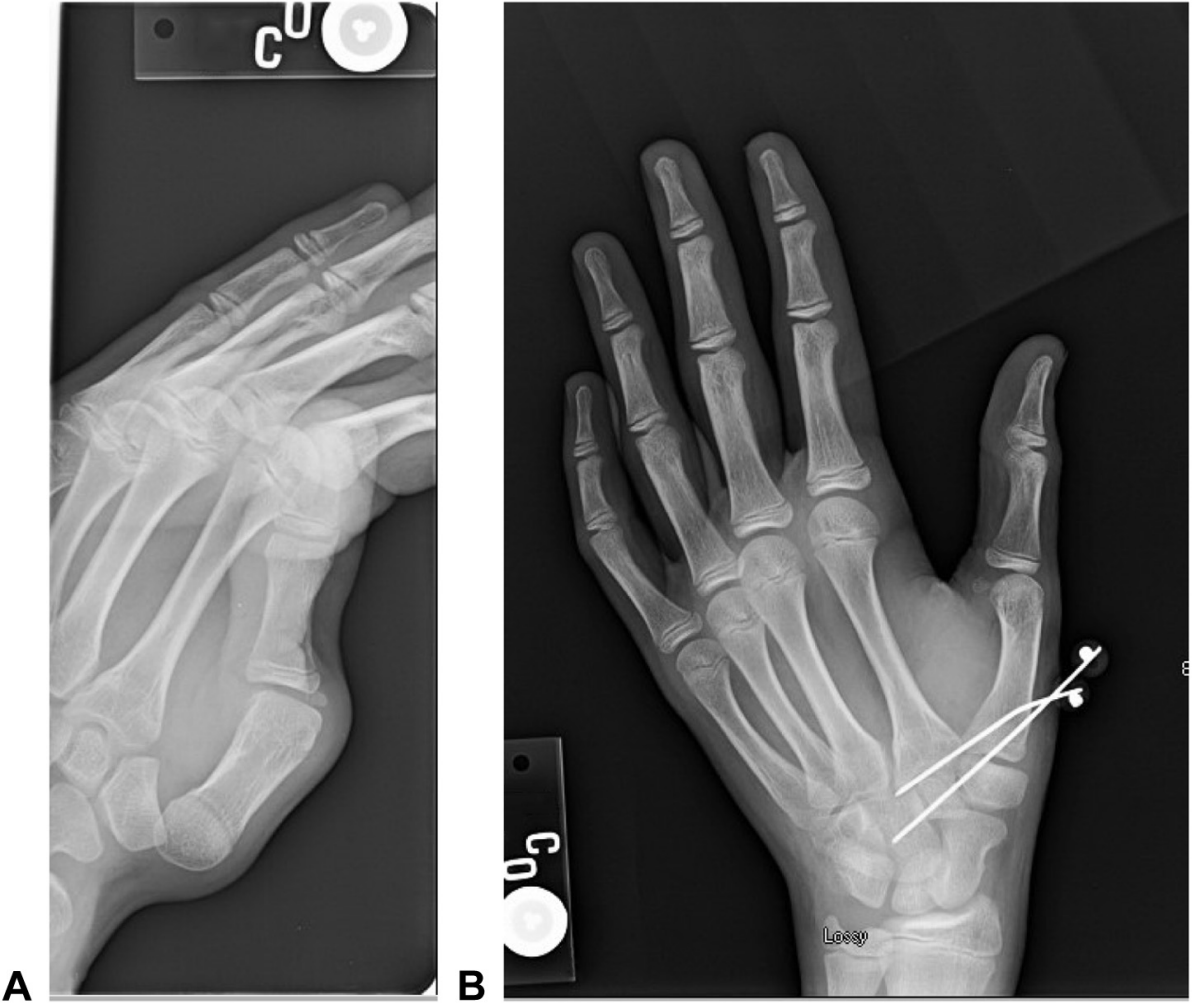
November 2013: Left CMC CRPP. The images represent the **A** preoperative and **B** postoperative alignments following the left CMC’s CRPP.

However, in November 2013, the patient dislocated his left CMC and metacarpophalangeal joints while pulling up his pants. He underwent closed reduction at the clinic, and magnetic resonance imaging was performed, demonstrating discontinuity in the dorsoradial ligament. He subsequently underwent CRPP of the left CMC (Fig. 3).

**Figure 4:**
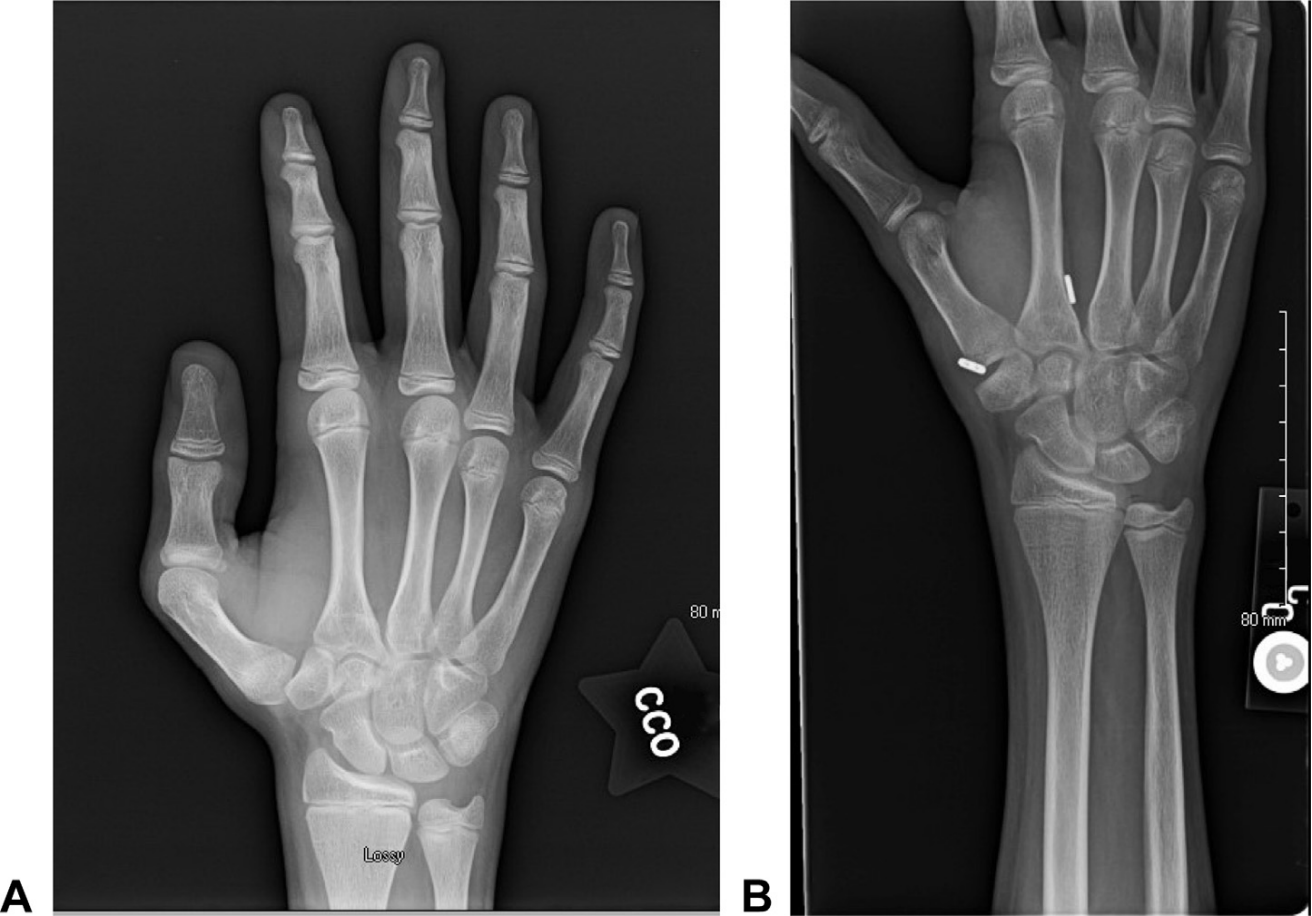
January 2014: Tightrope placement in the right hand. The images represent the **A** preoperative and **B** postoperative films of tightrope placement between the thumb metacarpal and index metacarpal in the right hand.

In January 2014, the patient once again dislocated his right CMC joint while pulling up his pants. Because of the failure of casting and CRPP, the decision to proceed with mini-tightrope stabilization of the right CMC joint using the Mini TightRope fixation system (Arthrex) was made (Fig. 4). Intraoperatively, there were no signs of subluxation, and the patient initially had good results following the procedure.

**Figure 5:**
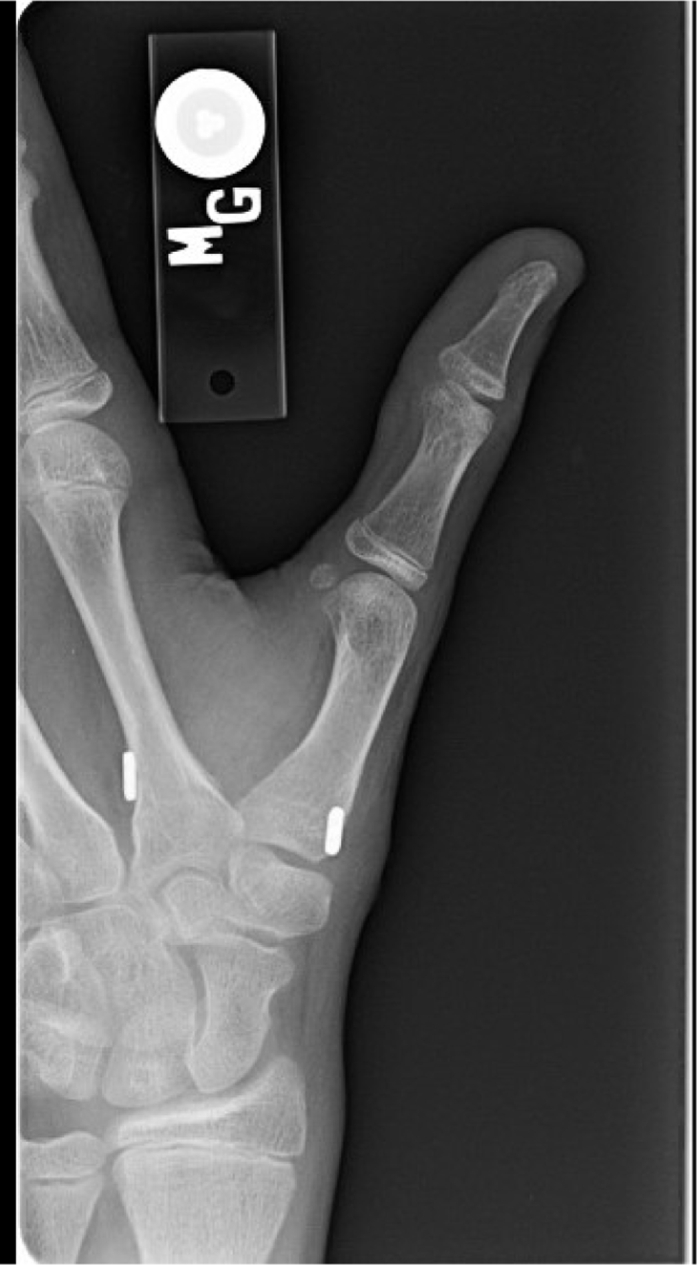
March 2014: Tightrope placement in the left hand. The image represents the postoperative film of tightrope placement between the thumb metacarpal and index metacarpal in the left hand.

In March 2014, he reinjured the left thumb while closing a shower bottle cap. Because of the recurrent instability, he underwent tightrope fixation on the left side (Fig. 5). After the surgery, the patient was doing well, when in July 2014, he sustained a baseball injury to the left thumb. Upon examination, the left thumb appeared to have subluxated in the palmar direction, with no evident dislocation. The tightrope construct appeared intact; so, the patient underwent closed reduction, and a spica cast was placed for 6 weeks. Unfortunately, he continued to experience chronic laxity and subluxation following this intervention.

In October 2014, the patient once again dislocated the right thumb, this time while playing video games. The patient underwent reduction, and a cast was fitted at the clinic. Magnetic resonance imaging demonstrated a well-reduced CMC joint while in the cast but also showed a rupture in the suture. The decision was made to further stabilize the right thumb’s CMC joint using repeat tightrope fixation with an abductor pollicis longus tendon graft to reconstruct the anterior oblique ligament.

At that time, skin samples were collected for collagen typing but were largely unrevealing because hypermobility-type Ehlers-Danlos syndrome cannot be detected using a genetic test and skin fibroblast typing has largely fallen out of favor and is not readily available for routine evaluation. Echocardiography was also performed for the concern of Marfan syndrome, with unremarkable results. Additionally, a clinical evaluation of the patient by our genetics clinic suggested that he did not have Marfan or Ehlers-Danlos syndrome, rather had a nonspecific connective tissue disorder.

Most recently, having experienced excellent outcomes after the partial abductor pollicis longus reconstruction of his right thumb, the patient elected to undergo the same procedure for his chronically lax left thumb. Following this surgery, he has not required further intervention.

**Figure 6:**
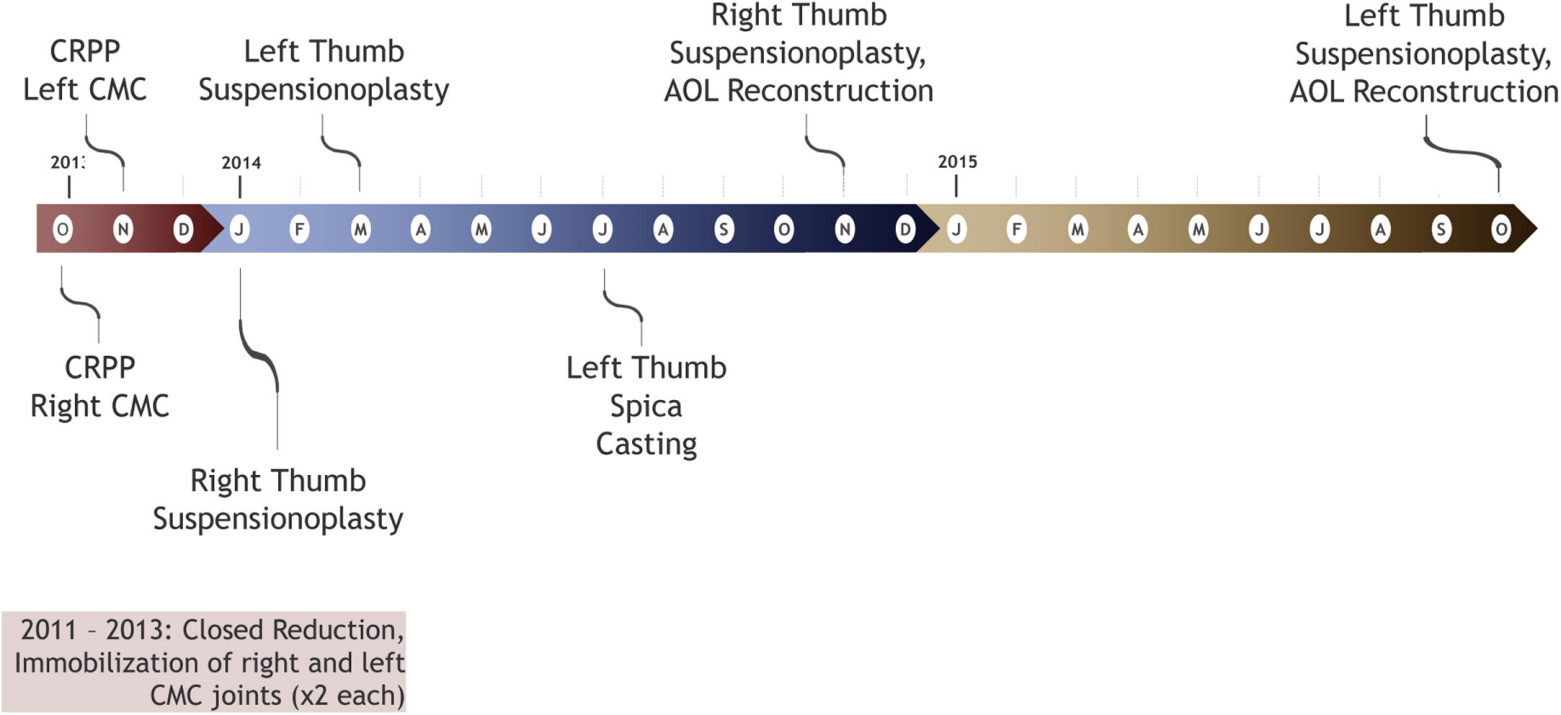
Treatment timeline. A graphical representation of the timeline of the patient’s treatment with procedures from October 2013 to October 2014.

In total, the patient underwent 10 procedures over a course of 4 years (Fig. 6). Each thumb underwent closed reduction and immobilization, which eventually failed, leading to CRPP being performed at our institution. After the failure of CRPP, each thumb underwent tightrope placement using the Mini TightRope fixation system (Arthrex), which eventually required a revision with an anterior oblique LR. Following these final procedures, the patient has done well over a course of 5 years of follow-up. He was recently seen at our clinic in June 2021. Strength and mobility testing was performed at that time, which demonstrated good range of motion and strength bilaterally (Table 1). He also completed the Quick Disabilities of the Arm, Shoulder and Hand questionnaire administered at that time, with a score of 2.3, suggesting an excellent functional outcome. He currently works as a mechanic and, aside from occasional aching with repetitive use, reports that his thumbs are doing well. He does not require orthoses or other supportive devices.

**Table 1:** Range of Motion and Strength Testing at 5-Year Postoperative Routine Follow-Up

Functional Measurement	Laterality	
	Right	Left
Strength (kg)		
Grip	52.1	47.6
Apposition	9.0	11.8
Opposition	10.4	10.4
Motion (degrees)		
Abduction	85°	95°
IP extension	10°H	10°H
IP flexion	35°	40°
MCP extension	10°	0°
MCP flexion	90°	80°

IP, interphalangeal; MCP, metacarpophalangeal.

## Discussion

An incompletely treated CMC dislocation can cause chronic instability, arthritis, and decreased grip strength.^3^ The literature on the treatment of these injuries in adults is mixed, but in the pediatric population, the outcomes are generally quite positive with prompt closed reduction.

In 1987, Watt et al^4^ reported a series of 12 adults with acute traumatic dislocations; 9 were treated with closed reduction and casting alone, and 3 were treated with CRPP. Six of the 9 patients treated with closed reduction and casting had satisfactory results compared with only 1 of the 3 patients treated with CRPP. Notably, all patients treated on the day of the injury had satisfactory results, whereas all patients with delayed presentation had some discomfort or instability, regardless of the treatment. The authors recommended a treatment algorithm of closed reduction with immediate joint stability assessment for patients with early presentation and CRPP for patients with a delayed presentation or with continued instability after early reduction. If the patients developed symptomatic instability later, they recommended LR. They emphasized the importance of assessing postreduction joint stability and posited that delayed treatment might increase long-term joint instability.

In 1996, Simonian et al^5^ compared retrospective cohorts of patients who underwent pinning of CMC dislocations with those who underwent early LR. None was treated solely with closed reduction and casting, and all had substantial instability at the time of closed reduction. In all cases of LR, there was disruption of the anterior oblique ligament. The patients in the CRPP group had increased pain, increased subluxation, and decreased range of motion compared with those in the LR group. Based on their results, they recommended early reconstruction in patients with instability because of the decreased pain as well as better range of motion and grip strength found in the LR cohort. In contrast to the study by Watt et al,^4^ none of these patients had immobilization prior to surgery, and none had stability after closed reduction; the timing to initial reduction was not reported.

In 2015, Lahiji et al^6^ reported a series of 6 dislocations, 5 treated with LR and 1 treated with closed reduction alone because of patient preference. All initially underwent reduction within 24 hours of their injury and had satisfactory outcomes, although postreduction stability was not reported.

The reports of CMC dislocation in the pediatric population are scarce. Nusem et al^7^ reported a case of a 10-year-old girl with a CMC dislocation, and Tyagi et al^8^ reported a case of traumatic floating first metacarpal (dislocation of the metacarpophalangeal and CMC joints), both of which were treated with early closed reduction and casting. Notably, Tyagi et al^8^ mentioned that their patient had no history of ligamentous laxity. In both the cases, the joint underwent reduction on the day of the injury; was confirmed stable at the time of reduction; and had full range of motion, without pain, stiffness, or instability at subsequent follow-up. Like Watt et al,^4^ Nusem et al^7^ attributed their good results to early treatment and the likely intact anterior oblique ligament.

Unlike these pediatric cases that had experienced trauma, which responded well to closed reduction and casting, our case of bilateral dislocation ultimately required an LR. This could have been due to multiple factors, including the delayed presentation and possible underlying ligamentous laxity, given the bilateral nature of his dislocations and the low-energy mechanisms of most of his injuries. Given our experience, it may be appropriate to consider proceeding with an aggressive operative intervention, including LR, in patients with either delayed presentation or injury patterns concerning nonspecific ligamentous laxity. If LR had failed, CMC arthrodesis would have been an appropriate salvage procedure. It has been shown to provide a high level of patient satisfaction and symptomatic relief in patients with chronic, painful laxity, and it might have been considered^9^ Ultimately, this case, in conjunction with the literature review, emphasizes early treatment and postreduction stability assessment, with LR, in the setting of long-term instability.
